# GATE Monte Carlo evaluation of cellular dosimetry for ^119^Sb in targeted radionuclide therapy

**DOI:** 10.1186/s40658-026-00884-2

**Published:** 2026-06-05

**Authors:** Arsalan Hasnaki, Nahid Chegeni, Barat Barati, Ghasemali Divband, Etesam Malekzadeh

**Affiliations:** 1 Cancer, Environmental and Petroleum Pollutants Research Center, Ahvaz Jundishapur University of Medical Sciences, Ahvaz, Iran; 2 Department of Medical Physics, Faculty of Medicine, School of Medicine, Ahvaz Jundishapur University of Medical Sciences, Ahvaz, Iran; 3 Department of Radiology Technology, Shoushtar Faculty of Medical Sciences, Shoushtar, Iran; 4 Nuclear Medicine Center, Jam Hospital, Tehran, Iran; 5 School of Medicine‚ Abadan University of Medical Sciences, Abadan, Iran

**Keywords:** Auger electron emitters, Cellular dosimetry, Micro dosimetry, Targeted radionuclide therapy, Monte Carlo simulation, S values, Gate

## Abstract

**Background:**

This study evaluates the cellular dosimetry of Antimony-119 (^119^Sb) as a candidate for targeted radionuclide therapy (TRT), using Monte Carlo simulations with the GATE toolkit. The goal is to assess the microdosimetric characteristics of ^119^Sb as an Auger and Internal conversion electron emitter and compare its performance with other clinically relevant radionuclides—^177^Lu, ^125^I, ^123^I, and ^103^Pd—across different cellular geometries and emission spectra.

**Methods:**

Monte Carlo simulations were performed using the GATE toolkit to model energy deposition at the cellular level. Two cell geometries were considered: one with a centrally located nucleus and another with the nucleus adjacent to the cell membrane, reflecting real biological diversity. S-values (absorbed dose per decay) were calculated for three possible locations of radioactivity (nucleus, cytoplasm, and cell membrane). Three emission spectra and two physics models were used to ensure robust results. Simulations were benchmarked against established dosimetry models (MIRDcell, PENELOPE, and Geant4).

**Results:**

^119^Sb delivered the highest and most localized dose to the cell nucleus, particularly when radioactivity was at the cell membrane, outperforming the other radionuclides tested. Its S-values were up to five times higher than ^177^Lu and four times higher than ^125^I in certain configurations. This effect remained strong even when the nucleus was off-center, suggesting ^119^Sb’s effectiveness is independent to nucleus locations diversities. Auger and Coster-Kronig electrons dominated the energy deposition for most isotopes in single cell configurations, while internal conversion electrons contributed significantly for ^119^Sb in the cytoplasmic and membrane configurations.

**Conclusions:**

^119^Sb is a promising candidate for TRT of micrometastases and single cancer cells due to its high and stable S-values, especially when radioactivity is localized at the cell membrane. Its dosimetric stability across nucleus positions makes it particularly suitable for therapeutic applications where cellular morphology varies. However, production and radiolabeling challenges may limit its clinical adoption.

**Supplementary Information:**

The online version contains supplementary material available at 10.1186/s40658-026-00884-2.

## Highlights

Antimony-119 (^119^Sb) delivers significantly higher and more localized radiation doses to cell nuclei than other tested radionuclides.

Monte Carlo simulations with GATE showed ^119^Sb’s effectiveness is robust, even when the cell nucleus is off-center.

The primary dose contribution for ^119^Sb comes from Internal Conversion electrons for N⇓CM.

^119^Sb shows strong potential for targeted radionuclide therapy of single cells.

**Supplementary Information:**

The online version contains supplementary material available at 10.1186/s40658-026-00884-2.

## Background

DNA is the most sensitive component of the cell to ionizing radiation, and the efficacy of a treatment protocol strongly depends on the extent of damage inflicted upon the nucleus and ultimately DNA. In External Beam Radiotherapy (EBRT), target volumes are uniformly exposed to radiation, resulting in a relatively homogeneous dose distribution across the nucleus and other organelles within the target cells. However, in targeted radionuclide therapy (TRT), the dose received by a cell’s organelles can vary significantly depending on the type of radiation, particle penetration range, and the distribution of radiopharmaceuticals around and within cells [1]. TRT was introduced several decades ago to treat thyroid cancer using Iodine-131 (^131^I). More recently, its potential has been explored with various other radionuclides, including beta (β), alpha (α), and Auger electron (AE) emitters. Lutetium-177 PSMA-617 was approved by the FDA for treating metastatic castration-resistant prostate cancer (mCRPC), based on the therapeutic efficacy of its beta emissions and high PSMA expression in this type of cancer [2]. While TRT is mainly used in advanced diseases for palliative treatments, its earlier application to eradicate disseminated tumor cells (DTCs) and occult micro-metastases may offer more effective treatment [3].

In many cancers, the risk of distant recurrence can be predicted based on clinicopathological and genomic features, response to neoadjuvant treatment, the presence of CTCs (Circulating Tumor Cells) or circulating tumor DNA, or other biomarkers [4]. To successfully prevent a recurrence, TRT must be capable of eradicating lesions of various sizes, including occult micro-metastases, single cells, and clusters of CTCs and DTCs. It remains unclear which isotopes are most suitable for adjuvant therapy, especially when the target tumors are undetectable by radiological examinations and are likely to be very small (ranging from micrometers for single tumor cells to lesions 5–10 mm in diameter). However, currently utilized radionuclides that emit medium-energy (^177^Lu, ^131^I) or high-energy (^90^Y) beta particles are suboptimal for the TRT of tiny tumor lesions, as the majority of their energy is deposited outside these lesions. Other radionuclides are being investigated, including more advantageous β emitters, α emitters, and AE emitters [5, 6].

In general, Auger electron (AE) emitters are highly recommended for treating single cancer cells or small micrometastases. By using a targeted carrier—such as a small molecule, peptide, or antibody—high levels of radiation can be delivered to sites characterized by tumor-specific biomarkers while simultaneously minimizing off-target toxicity in healthy cells [7]. AE-emitting radionuclides release a cascade of electrons with energies ranging from a few electron volts (eV) to several tens of kiloelectron volts (keV). Coster-Kronig (CK) and Super Coster-Kronig (SCK) transitions constitute a major portion of transitions of AE decays, so the yield of low-energy electrons per decay is typically higher magnitude than the yield of high-energy electrons. Due to their short penetration range in biological matter (from nanometers to micrometers), low-energy electrons are considered high Linear Energy Transfer (LET) particles (10–25 keV/µm) [8]. They cause highly localized energy deposition over small distances at cellular and subcellular scales [9, 10].

Another important consideration is the physical half-life of the radionuclide. As the physical half-life increases, the maximum achievable specific activity of a radionuclide decreases, potentially limiting the therapeutic efficacy due to lower radiation dose delivery to the target tissue. Moreover, a prolonged half-life increases the risk of the therapeutic agent redistributing throughout the body before delivering sufficient radiation to induce lethal damage to tumor cells. Therefore, selecting a radionuclide with an appropriate physical half-life is crucial to ensure optimal balance between effective tumor targeting and minimizing off-target toxicity. This balance is essential for maximizing therapeutic efficacy while reducing potential side effects [11, 12].

Antimony-119 (^119^Sb), a promising Auger and Coster-Kronig (CK) electron emitter, has a suitable half-life of 38.19 h, making it a potential candidate for TRT. Despite significant obstacles to using this radionuclide in medical trials and radionuclide therapy studies—such as limited production routes and challenges in developing effective radiolabeling methods, recent studies have been pursued to introduce ^119^Sb into clinical practice for cancer treatment. The ^119^Sn(p,n) ^119^Sb reaction has been identified as a most suitable method for ^119^Sb production, offering high specific activity, high production yield, and utilizing low-energy cyclotrons [13–15].

Given the microscopic dimensions involved, experimental dosimetry at the cellular or subcellular level remains unfeasible. Consequently, calculations remain the only way to evaluate the suitability of AE emitters for clinical applications. The most common method for internal dosimetry of radionuclides is via the Medical Internal Radiation Dose (MIRD)[16, 17] This model typically assumes a uniform distribution of radioactivity within defined source regions, such as specific cellular compartments, to calculate the mean absorbed dose in target regions. The MIRD Committee has developed a comprehensive set of S-values for spherical cells of various sizes, monoenergetic sources, and radionuclides, facilitating internal dosimetry calculations across a wide range of scenarios. These tabulated S-values have streamlined the process of estimating absorbed doses in source-target organ pairs. However, the MIRD formalism has inherent limitations, notably its inability to discern of contributions of each transition in deposited energy and its inability to accurately model the transport of low-energy electrons, such as Auger electrons. In fact, the MIRD approach assumes electrons travel in straight paths, without considering energy straggling. With advancements in computational capabilities, the Monte Carlo (MC) method has emerged as the gold standard for internal dosimetry in nuclear medicine. In general, MC simulations excel in modeling the stochastic nature of radiation interactions, enabling precise calculations of energy deposition at microscopic scales. This approach overcomes the simplifications of the MIRD schema by accounting for complex physical processes, including scattering, energy loss, angular deflections, and the production of secondary electrons (delta rays). Consequently, MC methods provide a more accurate representation of dose distributions, particularly at the cellular and subcellular levels, which is crucial for evaluating the therapeutic efficacy of radionuclides emitting low-energy electrons [8, 18, 19].

The purpose of this study is to calculate S-value and investigate cellular microdosimetry for ^119^Sb as a candidate for Auger electron therapy of micrometastases, and compare with ^177^Lu,^125^I, ^123^I, and ^103^Pd for two geometrical scenarios: one with the nucleus centrally located, and another with the nucleus adjacent to the cell membrane. We calculate S-values and deposited energy for different radiation components —Auger electrons, Coster-Kronig (CK) electrons, internal conversion (IC) electrons, and photons—using three different emission spectra and two distinct Monte Carlo physics models. All calculations are performed with the GATE Monte Carlo simulation toolkit, a gold-standard platform for cellular dosimetry in nuclear medicine.

## Methods

### Simulation by GATE code

**Fig. 1:**
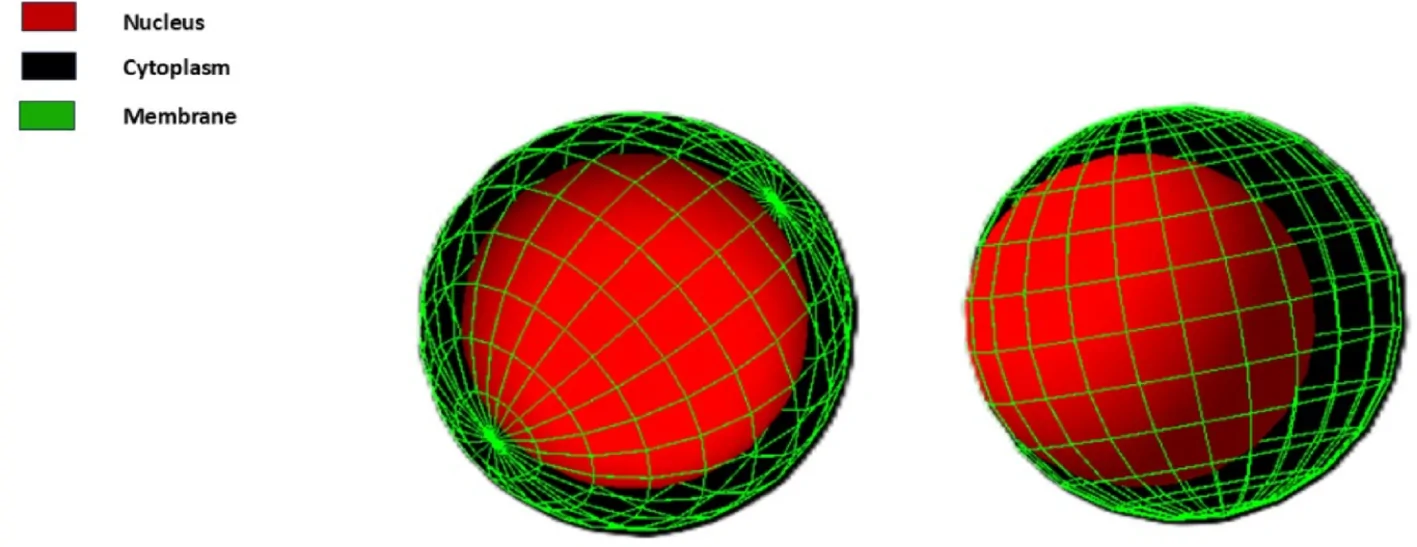
Single cell of 5µm radius with concentric nucleus of 4µm radius and 10nm cell membrane. (Left) Eccentric 4µm nucleus circumscribed by 10nm cell membrane within a 5µm cell. (Right)

The Monte Carlo code Geant4 Application for Tomographic Emission (GATE) was used to simulate two homogeneous concentric water-based spheres representing the overall cell geometry and the nucleus, along with a spherical shell as the cell membrane**.** As can be seen in Fig. 1, the sizes were determined based on simulations performed on a 5 µm cell and a 4 µm nucleus, which are reported sizes for typical lymphocyte cells [20] and Chinese hamster cells (V79) [21, 22]. For electrons, GATE offers several condensed-history electromagnetic (EM) models to facilitate the simulation of a large number of interactions. The physics models used in the simulations were also investigated in “emDNAPhysics” and “empenelope". GATE’s existing cut-off for physics is 8 eV and 100 eV, respectively, which makes this study more capable for comparing these models to each other, and previous studies as benchmarks [23]. The “world” of the simulation was chosen as cubic of “G4_WATER” with density 1 g/cm^3^ and dimensions 30 µm due to it being the range that 100% of all AE and CK spectra are stopped and absorbed in water [24].

### Cellular dosimetry

The absorbed dose for radionuclides was calculated based on the MIRD methodology [25]. According to calculations of the MIRD committee, the mean absorbed dose is described as:

1 $$\overline{D }\left(T\leftarrow S\right)={\widetilde{A}}_{s}\cdot S\left(T\leftarrow S\right)$$

The mean absorbed dose in a target area $\overline{D }\left(T\leftarrow S\right)$​ is determined by the total activity accumulated in the source area (${\widetilde{A}}_{s}$​), and the S-value $S\left(T\leftarrow S\right)$ represents the mean dose delivered to the target area per radionuclide disintegration in the source area, and is defined as follows:

2 $$S\left(T\leftarrow S\right)=\frac{1}{{m}_{t}}\sum_{i}{y}_{i}{E}_{i}{\phi}_{i}\left(T\leftarrow s\right)$$

Here, ${y}_{i}$​ represents the yield of electrons (number of electrons) emitted during a specific transition ‘*i*’. Each of these emitted electrons carries an energy of ${E}_{i}$, and ${\phi}_{i}$ represents the fraction of the source energy that is deposited within the target region, and ${m}_{t}$ is the mass of the target [26].

Utilizing the “Edep” actor available in the GATE toolkit, the deposited energy within the nucleus volume was computed, according to the formula proposed by Gardint et al. [27], This energy can be calculated as:

3 $$S\left(T\leftarrow s\right)=\frac{k}{{R}^{3}}\sum {y}_{i}{E}_{i}{\phi}_{i}\left(T\leftarrow S\right)$$

where *k* =3.82 cGy/keV, ${E}_{i}$ is in keV, and *R* corresponds to the cell or nucleus radius in μm. As considered, the term $\sum {y}_{i}{E}_{i}{\phi}_{i}\left(T\leftarrow S\right)$ denotes the deposited energy that is calculated by the “Edep” actor.

### Radionuclides and spectra

Five radionuclides, ^177^Lu, ^123^I, ^125^I, ^103^Pd, and ^119^Sb, were utilized in this study. ^177^Lu was selected for its significant clinical applications; the remaining radionuclides were chosen based on their abundant AE decay characteristics, which facilitated a direct comparison with ^119^Sb.

This study utilized three distinct beta spectra: MIRD Average β spectrum (Avg), MIRD Full β spectrum (Full), and AAPM spectrum. The Avg spectrum was derived using the MIRD-RADTABS v2.2 and MIRDcell v4.15 software following the methodology established by Eckerman and Endo [28]. The Full spectrum was extracted from MIRDcell v4.15, drawing upon MIRD monographs [25], and the AAPM spectrum was specifically chosen as the MC facilitated spectrum from AAPM data center [8]. To enable a more direct and effective comparison across all three spectral datasets, it is necessary to note that for ^123^I, ^125^I, three spectra are available; for ^177^Lu and ^103^Pd, Avg and Full MIRD spectra are available; whereas for ^119^Sb, only the Average MIRD spectrum is available.

To validate our findings, we conducted a benchmark using MIRDcell v4.15, which requires modeling two adjacent spherical cells (each 5 µm in radius with a 4 µm radius nucleus). Although this setup enables both self and cross-dose calculations, our comparisons focused exclusively on the self-dose component to align with the GATE-based S-value method. We computed self-dose S-values using both Avg and Full β spectra across the N ← N, N ← Cy, and N ← CM configurations [29]. Additionally, we validated our results against established studies by Falzone et al. [24] (used PENELOPE with Avg spectra) and Seifi Moradi et al. [19] (used Geant4 with AAPM spectra), providing robust cross-platform verification of our dosimetric approach.

We defined radionuclide emission spectra using GATE’s discrete “UserSpectrum” module, which allows importing detailed spectra for Auger + Coster-Kronig (AE + CK), internal conversion electrons (IC), β, and X-/γ-photons. For each component—AE + CK, IC, β, and photons— the deposited energy in the nucleus (N) was individually calculated with the “Edep” actor. These deposited energies were summed up to obtain the total energy deposition in the nucleus, assuming uniform activity distribution in the three source configurations: cytoplasm (N ← Cy), cell membrane (N ← CM), and nucleus (N ← N) [23].

We modeled the dose delivered to the cell nucleus in two geometrical scenarios: one with the nucleus centrally located, and another with the nucleus adjacent to the cell membrane. The simulations account for 1MBq activity within the nucleus, cytoplasm, and cell membrane, reflecting potential differences in radiopharmaceutical localization. The final phase of this study was simulations for an eccentric nucleus adhering to the cell membrane, and reported the corresponding results. It’s worth noting that the specific translation of 0.99 µm for simulating this eccentricity was chosen due to the nucleus’s proximity to the cell membrane, given its 10 nm thickness. The inclusion of ^177^Lu, a clinically established beta-emitter, and ^125^I, ^123^I, and ^103^Pd— well-known AE emitting radionuclides— allows a robust comparison with ^119^Sb, an Auger-, CK-, and conversion-electron emitter with promising therapeutic features.

## Results

### Calculation of S-value

Table 1 summarizes concentric cellular S-values for five radionuclides, computed using Avg, Full, and AAPM β spectra and two physics lists (emDNAPhysics and empenelope). To validate our results, we compared them against benchmarks derived from MIRDcell v4.15, the PENELOPE code [24], and Geant4 [19]. simulations finding shows the statistical uncertainty for 1 MBq activity remained below 2.7%, and photon contributions constituted less than 3% of total S-values, confirming that electrons dominantly contribute to cellular dosimetry in this model.

**Table 1:** S-values for a 4 µm nucleus concentrically positioned in a 5 µm cell across three geometries (N ← N, N ← Cy, N ← CM), computed using Avg, Full, and AAPM β spectra with both emDNAPhysics and emPenelope. Results are compared to benchmarks from PENELOPE, standard Geant4, and MIRD

Radionuclide	Spectrum	Physics of simulation	S-value (Gy/Bq.s) × 10^–4^																				
			(N ← N)							(N ← Cy)							(N ← CM)						
			Gate	MIRD ^b^	Diff (%)	Penelope ^c^	Diff (%)	Geant4 ^d^	Diff (%)	Gate	MIRD	Diff (%)	Penelope	Diff (%)	Geant4	Diff (%)	Gate	MIRD	Diff (%)	Penelope	Diff (%)	Geant4	Diff (%)
^123^I	Avg	emp^a^	30.9	28.7	7.6	29.7	4.0			3.53	2.68	31.7	3.05	15.1			2.35	1.35	74.0	1.91	23		
		emDNA	30.0		4.5		1.0			3.10		15.7		1.6			1.91		41.1		0.0		
	Full	emp	32.2	29.8	8.0					3.69	2.90	27.1					2.39	1.39	71.9				
		emDNA	31.1		4.3					3.19		10.1					1.99		43.2				
	AAPM	emp	31.5					29.1	8.2	3.60						34.1	2.45					2.21	10.9
		emDNA	30.6						5.1	31.0					2.76	10.2	2.03						-8.1
^125^I	Avg	emp	70.5	65.4	7.8	67.8	3.9			7.44	5.88	26.1	6.48	14.8			4.54	2.51	80.8	3.74	21.4		
		emDNA	68.4		4.5		0.8			6.51		10.7		0.4			3.69		47		-1.3		
	Full	emp	71.8	66.0	8.7					7.57	6.18	22.5					4.70	2.53	85.8				
		emDNA	69.4		5.1					6.58		6.4					3.73		47.4				
	AAPM	emp	58.2					53.3	9.1	7.10						39	4.97					4.40	13.1
		emDNA	56.4						5.8	6.03					5.10	18	3.97					4.40	-10.8
^177^Lu	Avg	emp	14.1	13.5	4.4					4.34	4.28	1.4					3.03	2.90	4.4				
		emDNA	13.8		2.2					4.24		-0.9					2.9		0.0				
	Full	emp	19.6	17.1	14.6					6.57	5.70	15.3					4.52	3.86	17.1				
		emDNA	18.3		7.0					6.16		8.0					4.21		9.07				
^103^Pd	Avg	emp	25.5	22.1	15.4					3.98	3.06	30.1					2.67	2.14	24.7				
		emDNA	24.2		9.5					3.69		20.6					2.54		18.6				
	Full	emp	25.7	22.2	15.8					4.01	3.13	28.1					2.66	2.14	24.3				
		emDNA	24.4		9.9					3.70		18.2					2.56		19.6				
^119^Sb	Avg	emp	80.6	61.5	31	71.6	12.6			22.5	14.2	58.5	19.7	14.2			16.8	10.6	58	14.9	12.8		
		emDNA	72.4		17.7		1.1			19.6		38		-0.5			14.9		40.6		0.0		

^a^ “emp” and “emDNA” refer to empenelope and enDNAPhyiscs, respectively

^b^ MIRD data taken from MIRDcell v4.15

^c^ Penelope code results taken from Falzone et al. [24]

^d^ Geant4 code results taken from Seifi Moradi et al. [19]

**Fig. 2:**
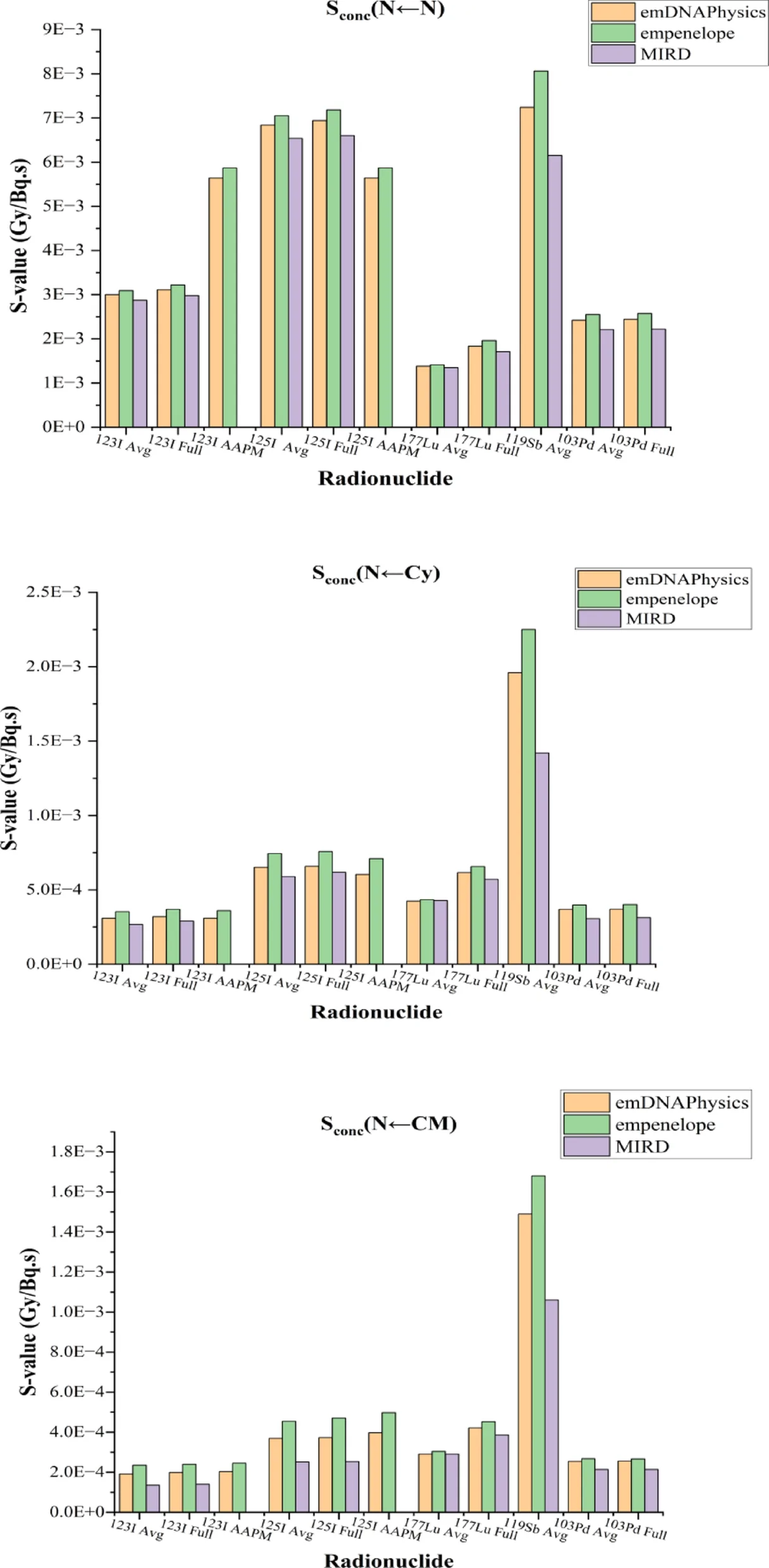
Exhibition of S-values based on “empenelope” and “emDNAPhysics” Gate calculations and comparison with MIRD for three spectra and three configurations, including N ← N, N ← Cy, and N ← CM

In the N ← N configurations, GATE Monte Carlo simulations using emDNAPhysics closely matched MIRDcell values, with deviation under 10% for all radionuclides except ^119^Sb. Specifically, the ^119^Sb S-value differed by only 1.1% compared to Falzone et al. (PENELOPE), whereas the empenelope result was 12.6% higher. When using the AAPM spectrum, results were also consistent (within ~ 5%) with Geant4-based data from Seifi Moradi et al. Figure 2 provides an exhibition of all S-values calculated using GATE for comparisons with MIRD.

For N ← Cy, deviation from MIRDcell increased—about 20% for emDNAPhysics and 30% for empenelope. Notably, ^119^Sb showed larger discrepancies (38% and 58.5% respectively), yet remained closely aligned with Falzone et al. (− 0.5% and 14.2%).

The N ← CM configuration exhibited the greatest differences to MIRD, especially with emDNAPhysics (~ 50%) and empenelope (~ 85%) for ^125^I with the Full spectrum (see Table 1). These discrepancies likely stem from MIRD’s assumption of straight-path electron travel without energy straggling. In most cases, GATE tends to overestimate S-values, except for ^177^Lu, which remains closely aligned with MIRD estimates, particularly when using emDNAPhysics. These variations are visually summarized in Fig. 2, illustrating the range of agreements and departures across the examined configurations.

### Effect of eccentricity

**Fig. 3:**
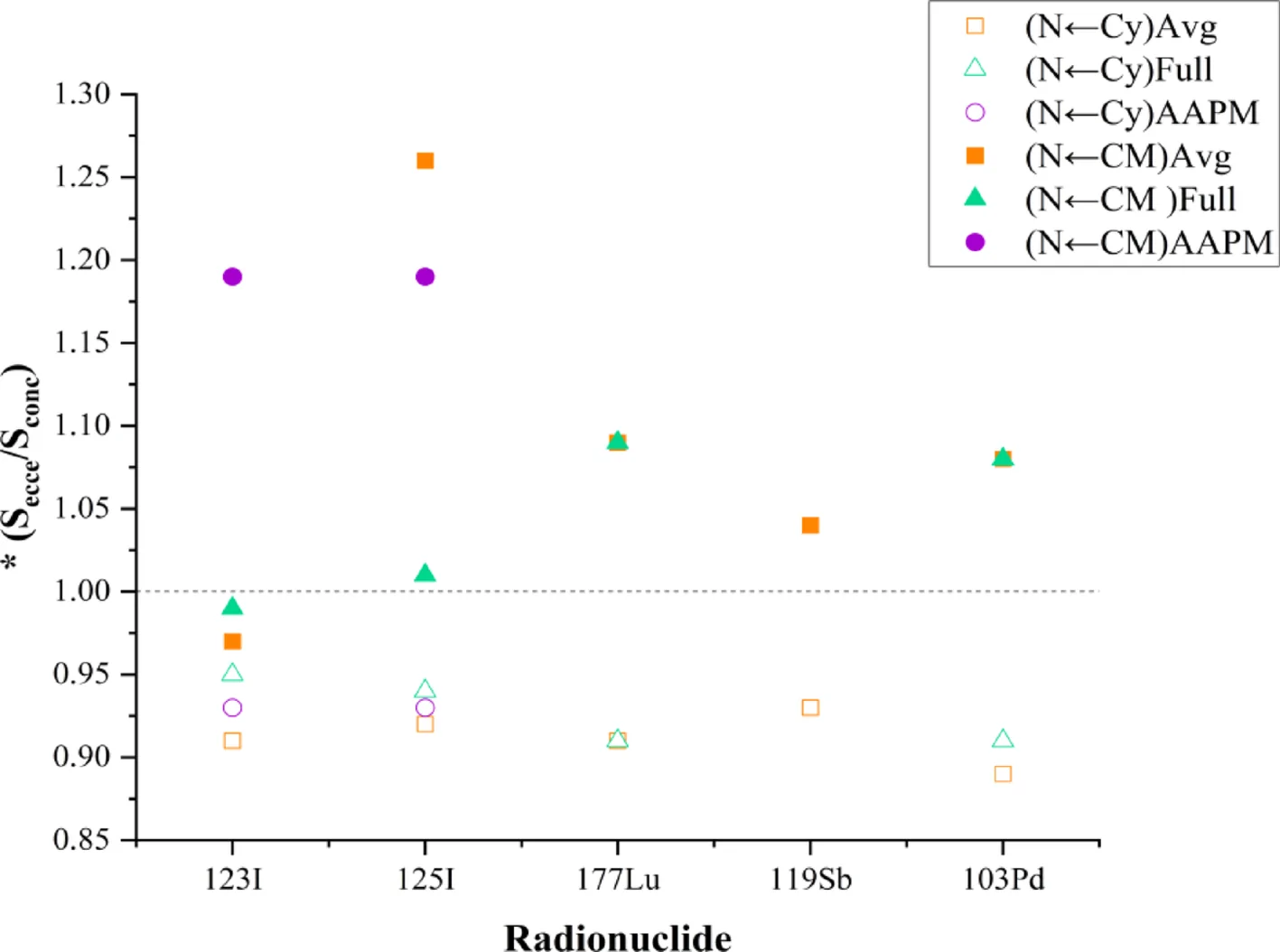
Schematic illustration of relative S-values for three spectra and two configurations. Full and Avg spectra show overlapping for some configurations. *(S_ecce_/S_conc_) is (S_eccentric_/S_concentric_) respectively

**Fig. 4:**
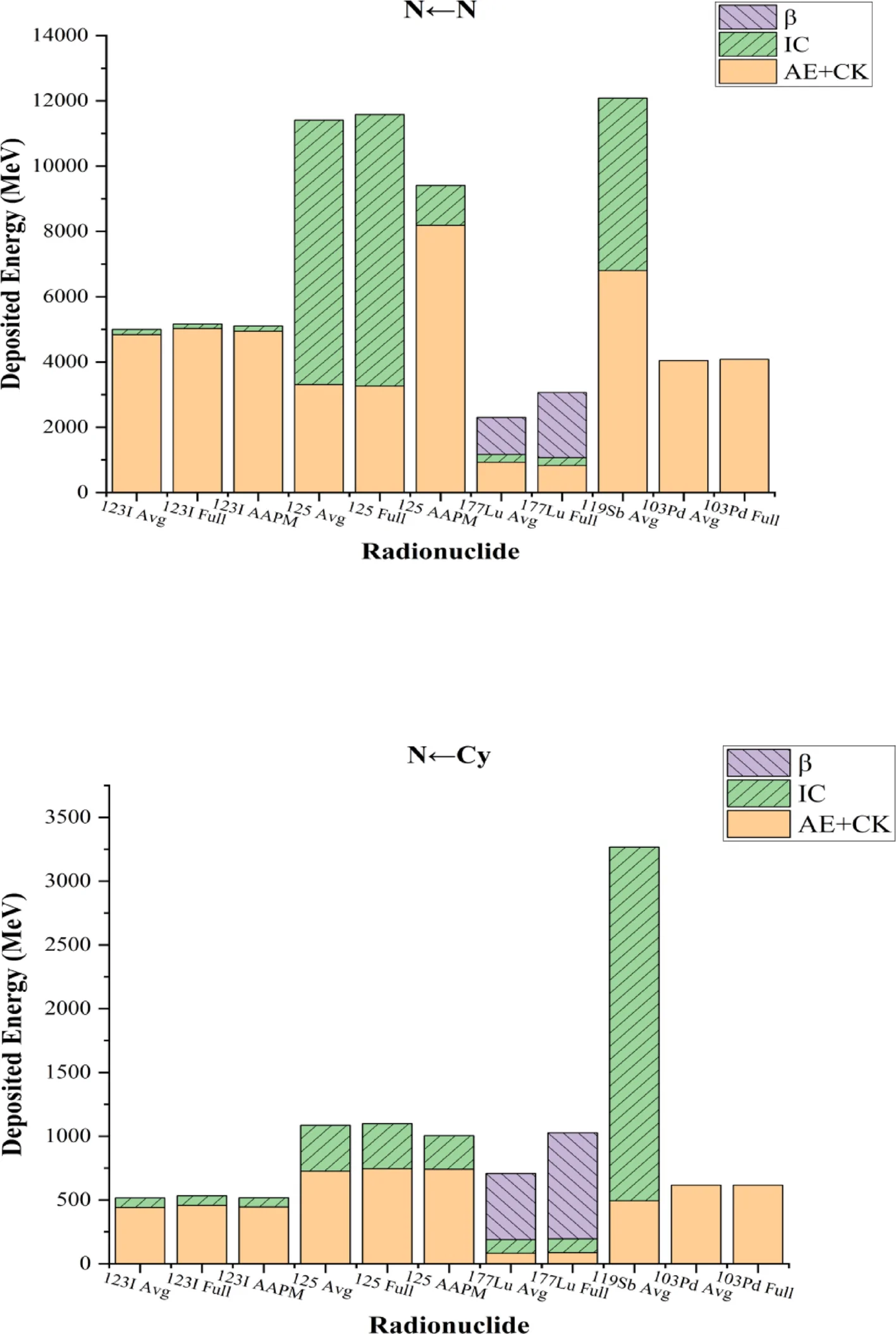

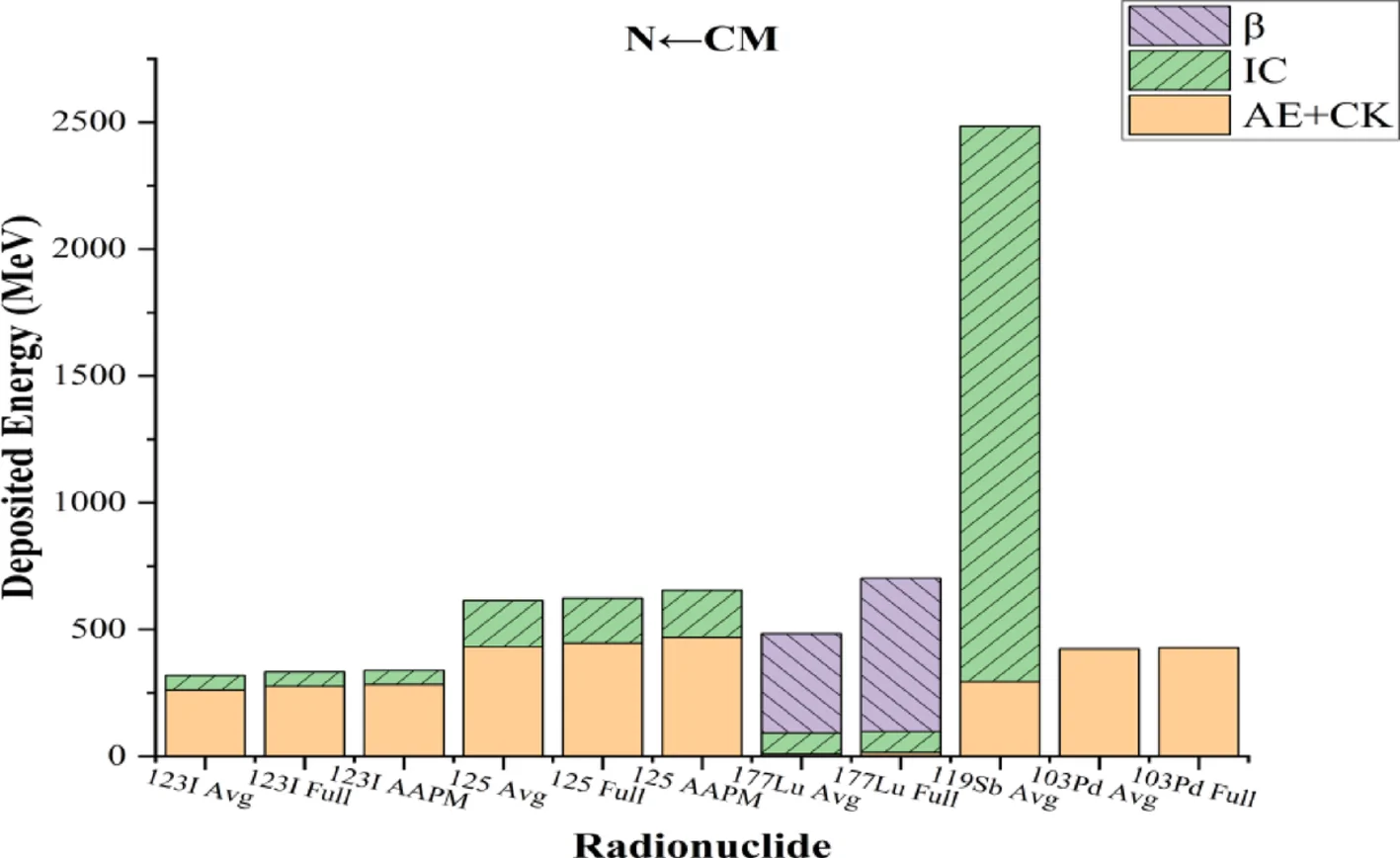
Contributions for all electron Transitions, including AE + CK: Auger and Coster-Cronig Electrons, IC: Internal Conversion electrons, and β for N ← N, N ← Cy, and N ← CM configurations

The influence of eccentricity on the calculated S-values is depicted in Table 2 and Fig. 3. As expected, the self-dose to the nucleus remains unaffected by its spatial position within the cell, whether centrally located or shifted toward the periphery. Overall, in the N ← Cy configuration, S-values consistently decrease by approximately 5–10% than concentric S-values across all radionuclides, due to reduced overlap between the source and the nucleus (Fig. 4).

**Table 2:** Eccentric S-values and their comparison with concentric results for two configurations N ← Cy, and N ← CM and, three spectra

		S-value (Gy/Bq.s) × 10^–4^					
		(N ← Cy)			(N ← CM)		
Radionuclide	Spectrum	eccentric	concentric	S_ecce_/S_conc_	eccentric	concentric	S_ecce_/S_conc_
^123^I	Avg	2.82	3.10	0.91	1.86	1.91	0.97
	Full	3.04	3.19	0.95	1.99	1.99	0.99
	AAPM	2.90	3.10	0.93	2.43	2.03	1.19
^125^I	Avg	5.99	6.51	0.92	4.65	3.69	1.26
	Full	6.25	6.58	0.94	3.76	3.73	1.01
	AAPM	5.64	6.03	0.93	4.69	3.97	1.19
^177^Lu	Avg	3.87	4.24	0.91	3.17	2.42	1.09
	Full	5.61	6.16	0.91	4.62	4.21	1.09
^103^Pd	Avg	3.31	3.69	0.89	2.74	2.54	1.08
	Full	3.39	3.70	0.91	2.78	2.56	1.08
^119^Sb	Avg	18.3	19.6	0.93	15.5	14.9	1.04

In contrast, when the radionuclide is positioned on the cell membrane (N ← CM), the S-values generally increase in comparison to the concentric configuration, although the magnitude of this increase varies among isotopes and spectral modes. For iodine isotopes, these gains are most pronounced**:** ^123^I with AAPM spectrum shows a 19% rise, while Avg and Full spectra are nearly equivalent to concentric values. Similarly, ^125^I exhibit a 26% increase using the Avg spectrum and 19% with AAPM, whereas the Full spectrum leads to only a 1% rise. In the case of ^177^Lu and ^103^Pd, both Avg and Full spectra yield increases of approximately 9% and 8%, respectively, and ^119^Sb shows a modest 4% increase with its Avg spectrum. While these differences are notable, they remain under 10% for most isotopes—consistent with prior Monte Carlo findings, which report similar ranges for eccentric configurations in nanoscale models.

### Contribution of transitions

Table 3 shows the contribution of Auger and Coster-Kronig (AE + CK) electrons, internal conversion electrons, and β-particles to total energy deposited in the cell nucleus (Edep) for each of the five diagnostic radioisotopes, across three concentric source-target configurations. In the N ← N configuration, Auger and CK emissions dominate the deposited energy for most spectra and isotopes, with the notable exception of ^177^Lu and ^125^I under Avg and Full spectra, where β and IC electrons contribute significantly. Specifically, ^177^Lu, a well-known β-emitter, exhibits an expectedly high β-electron contribution. The case of ^125^I reveals a considerable discrepancy: approximately 70–72% of energy deposition in the N ← N configuration arises from Internal Conversion electrons under both Avg and Full spectra, while using the AAPM spectrum reduces this IC share dramatically to 13%. This strong dependence on the chosen spectral model highlights the importance of spectral accuracy, particularly for isotopes with complex decay schemes such as ^125^I.

**Table 3:** Contributions of transitions for three spectra and three configurations

Radionuclide	Spectrum	Transitions	Deposited Energy in the cell nucleus, MeV		
			N ← N (%)	N ← Cy (%)	N ← CM (%)
^123^I	Avg	AE + CK	4835 (96)	441 (85)	261.7 (82)
		IC	166 (4)	76 (15)	56.7 (18)
	Full	AE + CK	5026 (96)	457 (85)	276 (83)
		IC	131 (4)	75.5 (15)	57 (17)
	AAPM	AE + CK	4941 (97)	445 (85)	445 (85)
		IC	161 (3)	73 (15)	73 (15)
^125^I	Avg	AE + CK	3307 (29)	725 (66)	431.9 (70)
		IC	8098 (71)	360.5 (34)	182.7 (30)
	Full	AE + CK	3262 (28)	744.5 (67)	445.7 (71)
		IC	8320 (72)	353.5 (33)	177.2 (29)
	AAPM	AE + CK	8182 (87)	740 (73)	468 (71)
		IC	1221 (13)	264 (27)	186.2 (29)
^177^Lu	Avg	AE + CK	925 (41)	81.4 (11)	9.5 (2)
		IC	240 (10)	107.7 (16)	81.9 (17)
		β	1137 (49)	518 (73)	392 (81)
	Full	AE + CK	829 (27)	87 (8)	15.1 (2)
		IC	240 (8)	108 (11)	81.8 (12)
		β	1994 (65)	832 (81)	605.7 (86)
^103^Pd	Avg	AE + CK	4039.6 (99.99)	615.8 (99.99)	423.32 (99.99)
		IC	0.4 (0.01)	0.01 (0.01)	0.01 (0.01)
	Full	AE + CK	4083.02 (99.99)	616.42 (99.99)	422.59 (99.99)
		IC	0.04 (0.01)	0.02 (0.01)	0.01 (0.01)
^119^Sb	Avg	AE + CK	6797 (56)	493.2 (15)	294.1 (11)
		IC	5285 (44)	2772.8 (85)	2190 (89)

For the N ← Cy and N ← CM configurations, Auger and CK electrons generally dominate over internal conversion (IC) and β electrons. However, exceptions are seen in the Avg and Full spectra of both ^177^Lu and ^119^Sb, where IC electrons contribute approximately 85% in N ← Cy and 89% in N ← CM. This highlights the substantial role of Internal Conversion in ^119^Sb’s dose deposition for these configurations. Remarkably, ^103^Pd exhibits nearly 99.99% contribution from Auger/CK electrons across all spectra and configurations.

## Discussion

Precise single-cell dosimetry is essential for optimizing the therapeutic efficacy of Auger electron-emitting radionuclides. In this study, we employed the GATE Monte Carlo toolkit (Geant4-based) to simulate detailed energy deposition events using complete emission spectra for each radionuclide. ur calculated cellular S-values demonstrated strong agreement with MIRD reference data when the source and target regions overlapped. Specifically, deviations were under 10% using the emDNAPhysics physics list and up to 20% with empenelope.

However, as spatial separation between the source and the nucleus increased—particularly in the nucleus-to-cell membrane (N ← CM) configuration—larger discrepancies were observed. The most pronounced deviation (85%) occurred when activity was localized within the cell membrane for the Full emission spectrum of ^125^I. These differences are largely attributed to the approximate MIRD methodology, which rely on collision stopping powers and electron range assumptions. This simplification presumes rectilinear electron trajectories, neglects energy straggling, and probably introduces systematic biases rather than statistical uncertainties [24, 30].

Among the radionuclides studied, ^119^Sb exhibits the highest S-values— 513% more than ^177^Lu (as a high usage clinical agent) and 403% more than ^125^I (as greatest value after ^119^Sb) in the N ← CM configuration (dominant configuration in TRT) with Avg spectrum and “emDNAPhysics” model.

The notably higher S-values observed for ^119^Sb, particularly for N ← Cy and N ← CM, stem from its greater yield of electrons with ranges comparable to cellular structures, as detailed in the supplementary information (Fig. S1-15). these electrons have microdosimetric ranges that extend effectively across cellular dimensions, they may enhance energy deposition in target structures. We further validated our S-value calculations using two key benchmarks, including Falzone et al. [24] who employed the PENELOPE code with the Avg spectrum under a 50eV cut-off, and Seifi Moradi et al. [19] whose Geant4 simulation utilized the AAPM spectrum. Despite their reliance on DPKs (Dose Point Kernel)) and higher cut-off threshold, our GATE simulations with emDNAPhysics —which uses a lower 8 eV cut-off— achieved excellent agreement with Falzone’s results [24] with discrepancies under 5% for all configurations. Comparisons with Seifi Moradi’s Geant4 data [19] show similarly high agreement in the N ← N and N ← CM configurations. In the N ← Cy configuration, emDNAPhysics results diverged by up to 18%, while empenelope exhibited larger discrepancies (39%), likely due to differences in energy cut-off and physics modeling. These inconsistencies may also point to limitations in the AAPM spectrum, especially for isotope ^125^I. Although, it is notable these studies have used different world of simulations, Geant4 Seifimoradi’s study used a cubic geometry with 12 µm world and GATE code used in this study using 30 µm world.

According to the superior agreement achieved with emDNAPhysics, particularly in eccentric geometries, all subsequent calculations—such as eccentric S-values and transition contributions—are based on this physics list. While the results obtained with the MIRD Avg and Full spectra showed close agreement, the AAPM spectrum yielded significantly different outcomes. This divergence starkly highlights the substantial impact of spectral model choice on cellular S-values, underscoring it as a critical source of uncertainty in microdosimetric calculations. The one exception was ^125^I under the N ← CM eccentric configuration, which showed greater divergence when using the Avg spectrum. It could be caused by the short range of its dominant low-energy electrons, making dose highly sensitive to geometric displacement. suggesting that spectrum choice may have a more pronounced impact in this specific case however, determining which spectrum is truly closest to physical reality would require further dedicated studies in cellular dosimetry and radiobiological microdosimetry. Our results indicate that ^119^Sb exhibits remarkably stable S-values across eccentric nucleus positions. Results in addition to showing agreements between Avg and Full spectra, represent that ^119^Sb,^123^I, and only the Full spectrum of ^125^I have less changes to the eccentricity of the nucleus. When comparing eccentric to concentric configurations (S_ecce_/S_conc_), ^119^Sb shows a − 7% change in N ← Cy and a modest + 4% change in N ← CM, demonstrating that eccentricity has minimal effect on its cellular dosimetry. This stability may be potentially advantageous, as natural variation in nucleus positioning would unlikely compromise therapeutic accuracy when using any radionuclide as a TRT agent. These findings align closely with Falzone et al. [24], who reported S_ecce_/S_conc_ of − 8% for cytoplasmic eccentrically and + 3% for membrane-localized activity with ^119^Sb. Their results were obtained using PENELOPE with DPKs and assuming a cell surface instead of a thin volumetric membrane (≈10 nm)—further underscoring the robustness and consistency of ^119^Sb dosimetry under various modeling assumptions. The observed differences between eccentric and concentric S-values for ^125^I (particularly the large N ← CM discrepancy from the average spectrum) may arise from its emission spectrum, which features lower-energy electrons with substantially higher yields compared to other radionuclides. Consequently, the dose ratio (eccentric-to-concentric) is more pronounced, as these electrons deposit energy preferentially in eccentric configurations.

As shown in Table 3, Auger and CK electrons dominate energy deposition rather than other transitions for most of the spectra. Specifically, in N ← N configuration, AE and CK emissions account for the majority of the dose. However, for N ← Cy and N ← CM cases, internal conversion electron (IC) contributes significantly more, reflecting the proximity of the source to the nucleus, indicating that ^119^Sb behaves predominantly as an IC emitter in scenarios typical of therapeutic applications, because radiopharmaceutical distribution rarely reaches the nucleus and consequently N ← Cy and N ← CM are dominant configurations.

A key obstacle to the clinical adoption of ^119^Sb has been the difficulty in producing sufficient quantities with high purity. Traditional methods, such as proton bombardment of enriched ^119^Sn targets, have limited scale and availability. However, Bennett et al. [31] introduced a scalable method utilizing high-energy proton irradiation of a natural antimony target (^nat^Sb) to produce ^119m^Te, which decays to ^119^Sb. This approach allows large-scale (curie-level) production with rapid separation of the ^119^mTe/^119^Sb pair [31]. More recently, Olson et al. [32] reported sustainable, cost-effective production via both (p,n) and (d,n) reactions on tin targets. Their method achieved radioisotopic purity better than 99.8%, with efficient chemical separation and significant target material recycling [32].

Although our study demonstrates that GATE with emDNAPhysics achieves strong agreement with reference models and benchmarks, several limitations must be considered. First and foremost, our single cell geometry limits direct extrapolation to multicellular and microtumor scales, where intercellular cross-irradiation and clustering effects significantly influence dosimetry. Second, the geometry is idealized—using uniform, concentric spheres, which may not fully capture the complex morphology of actual cancer cells. Third, we did not account for stochastic variations in radionuclide clustering, decay pathways, or intra-nuclear redistribution—features that real cells exhibit and that can impact microdosimetry outcomes. Forth, the evaluation used a single cell size with a relatively large nucleus-to-cell ratio, which may have influenced the results, particularly in eccentric configurations and low-energy β emitters [33]. Because our model used only one geometry, the impact of eccentric nuclear positioning on S-values may not fully capture the variability that would occur across different cell types. The eccentricity study was not intended to comprehensively address cellular geometries but rather to build upon the specific configuration used in prior published works (Falzone et al. and SeifiMoradi et al., who employed this geometry with different simulation codes) for direct comparability. Future work should investigate a range of cell and nuclear sizes to better assess the generalizability of our findings under more biologically representative conditions.

## Conclusions

Among the radionuclides studied, ^119^Sb exhibits the highest S-values for all noted configurations with Avg spectrum. Using “emDNAPhysics” model with 8eV cut-off demonstrated great agreement even utilizing general volume dosimetry with other benchmarks. Importantly, its S-value remains stable regardless of nucleus eccentricity, maybe potentially making ^119^Sb particularly reliable for therapeutic applications where nuclear positioning varies, however production challenges may remain a barrier to clinical use.

## Supplementary Information

Below is the link to the electronic supplementary material.

Supplementary Material 1

## Data Availability

All data generated or analyzed during this study are included in this published article.

## Abbreviations

TRT: Targeted Radionuclide Therapy
DNA: Deoxyribonucleic acid
FDA: Food and Drug Administration
AE: Auger Electron
CK: Coster-Kronig
SCK: Super Coster-Kronig
IC: Internal Conversion
CE: Conversion Electron
DTC: Disseminated Tumor Cell
CTC: Circulating Tumor Cell
LET: Linear Energy Transfer
GATE: Geant4 Application for Tomographic Emission
natSb: ^Natural^Sb (natural Antimony)
MIRD: Medical Internal Radiation Dose
MC: Monte Carlo
EM: Electromagnetic
