# Supplementary material for: GATE Monte Carlo evaluation of cellular dosimetry for 119Sb in targeted radionuclide therapy
Source: EJNMMI Phys. 2026 Jun 5;13:86. doi: 10.1186/s40658-026-00884-2 (PMC13493738; doi:10.1186/s40658-026-00884-2)
Supplement: Supplementary file 1 [file 40658_2026_884_MOESM1_ESM.docx]

**Supplementary:**

**Radionuclides spectra:**

Five radionuclides, ^177^Lu, ^123^I, ^125^I, ^103^Pd, and ^119^Sb, were utilized in this study. ^177^Lu was selected for its significant clinical applications; the remaining radionuclides were chosen based on their abundant AE decay characteristics, which facilitated a direct comparison with ^119^Sb.

This study utilized **three distinct beta spectra**: MIRD Average β spectrum (Avg), MIRD Full β spectrum (Full), and AAPM spectrum. The **Avg spectrum** was derived using the MIRD-RADTABS v2.2 and MIRDcell v4.15 software following the methodology established by Eckerman and Endo (1). The **Full spectrum** was extracted from MIRDcell v4.15, drawing upon MIRD monographs(2,3), and the **AAPM spectrum** was specifically chosen as the MC facilitated spectrum from AAPM data center (4). To enable a more direct and effective comparison across all three spectral datasets, it is necessary to note that for ^123^I, ^125^I, three spectra are available; for ^177^Lu and ^103^Pd, Avg and Full MIRD spectra are available; whereas for ^119^Sb, only the Average MIRD spectrum is available.

These graphs have extracted from MIRD-RADTABS v2.2 following the methodology of the **Avg spectrum** established by Eckerman and Endo(1) All graphs have shown based on energy of each transition in x-axis and yield per nuclear transformation in y-axis. The data for each energy based on yield per nuclear transformation has been extracted for using in Gate Monte Carlo discrete "UserSpectrum" module(5) (Supplementary figure 1 to figure 15). Falzone et al. as one of benchmarks used in this study, had utilized this spectrum (Penelope benchmark used this spectrum)(6)

**^119^Sb Avg Spectrum:**

**Supplementary Figure 1.** AE and IC electron spectrum for 119Sb

**Supplementary Figure 2.** AE+CK spectrum for ^119^Sb

Figure 2 shows the AE+CK spectrum of ^119^Sb. The AE+CK data have been integrated into the spectrum presented in Figure 1. In all simulations performed for all radionuclides based on this spectrum, the AE+CK data, similar to those in Figure 2, provided identical deposited energy to the AE+CK data of Figure 1. For example, the variation in deposited energy to the nucleus between the spectrum of Figure 2 and the AE+CK spectrum of figure 1 was less than 0.1%.

**Supplementary Figure 3.** X-ray and Gamma ray spectrum for ^119^Sb

Supplementary Figure 3 shown X-ray and Gamma ray spectrum for ^119^Sb. The share of all photons in deposited energy was less than 3% of total deposited energy, confirming which electrons dominantly contribute to cellular dosimetry in this model.

**^125^I** **Avg Spectrum:**

**Supplementary Figure 5**. AE+CK spectrum for ^125^I

**Supplementary Figure4**. AE and IC electron spectrum for ^125^I

**Supplementary Figure 6**. X-ray and Gamma ray spectrum for ^125^I

**^123^I** **Avg Spectrum:**

**Supplementary Figure 7.** AE and IC electron spectrum for ^123^I

**Supplementary Figure 9**. X-ray and Gamma ray spectrum for ^123^I

**Supplementary Figure 8**. AE+CK spectrum for ^123^I

**^177^Lu Avg Spectrum:**

**Supplementary Figure 10.** AE and IC electron spectrum for ^177^Lu

The supplementary figure 11, β electron Avg spectrum, is a discrete source data in MIRDcell while MIRD-Tab shown a continuous curve for this spectrum, we used discrete, for using identical source to MIRDcell, and comparing data with it.

**Supplementary Figure 11**. β electron spectrum for 177Lu

**Supplementary Figure 12.** X-ray and Gamma ray spectrum for ^177^Lu

**^103^Pd Avg Spectrum:**

**Supplementary Figure 13.** AE and IC electron spectrum for ^103^Pd

**Supplementary Figure 14**. AE+CK spectrum for ^103^Pd

**Supplementary Figure 15.** X-ray and Gamma ray spectrum for ^103^Pd

**AAPM spectra:**

The AAPM reference spectra provide data for the radionuclides 123I and 125I(4,7), but do not include spectra for 103Pd, 177Lu, and 119Sb. Seifi Moradi et al.(8) as one of benchmarks used in this study, had utilized this spectrum. (Geant4 benchmark used these spectrum).

| **^123^I** | | |
| --- | --- | --- |
| **Transision** | **E_i_** (keV) | **n_i_** (Yeild per nuclear transformation) |
| CK NNX | 0.0298 | 2.1 |
| Auger NXY | 0.0325 | 6.54 |
| CK MMX | 0.127 | 0.869 |
| CK LLX | 0.213 | 0.156 |
| Auger MXY | 0.461 | 1.97 |
| Auger LMM | 3.04 | 0.751 |
| Auger LMX | 3.66 | 0.202 |
| Auger LXY | 4.28 | 0.013 |
| Auger KLL | 22.4 | 0.0838 |
| Auger KLX | 26.3 | 0.0388 |
| Auger KXY | 30.2 | 0.0035 |
| IC 1 K | 127 | 0.13 |
| IC 1 L | 154 | 0.0179 |
| IC 1 M,N... | 158 | 0.0053 |

| **^125^I** | | |
| --- | --- | --- |
| **Transision** | **E_i_** (keV) | **n_i_** (Yeild per nuclear transformation) |
| CK NNX | 0.0299 | 3.51 |
| Auger NXY | 0.0324 | 10.9 |
| CK MMX | 0.127 | 1.44 |
| CK LLX | 0.219 | 0.264 |
| Auger MXY | 0.461 | 3.28 |
| Auger LMM | 3.05 | 1.25 |
| IC 1 K | 3.65 | 0.191 |
| Auger LMX | 3.67 | 0.34 |
| Auger LXY | 4.34 | 0.0211 |
| Auger KLL | 22.4 | 0.138 |
| Auger KLX | 26.4 | 0.059 |
| Auger KXY | 30.2 | 0.0065 |
| IC 1 L | 30.6 | 0.11 |
| IC 1 M, N… | 34.1 | 0.0284 |

**MIRD Full β spectrum:**

The **Full spectrum** was extracted from MIRDcell v4.15, drawing upon MIRD monographs. The **Full spectrum doesn’t include ^119^Sb.**

| **^125^I** | | |
| --- | --- | --- |
| **E_i_ (MeV)** | **n_i_** | **Transition** |
| 5.578E-05 | 0.62224 | AE |
| 0.0006988 | 2.9915 | AE |
| 0.0029555 | 0.63613 | AE |
| 0.0032261 | 0.27737 | AE |
| 0.0035533 | 0.09345 | AE |
| 0.0036781 | 0.80029 | IC |
| 0.0037197 | 0.3262 | AE |
| 0.0037693 | 0.05521 | X-ray |
| 0.0039903 | 0.13518 | AE |
| 0.0040295 | 0.035828 | X-ray |
| 0.004252 | 0.04634 | AE |
| 0.0043016 | 0.009938 | X-ray |
| 0.0043175 | 0.055827 | AE |
| 0.0045226 | 0.019132 | AE |
| 0.0048498 | 0.007799 | AE |
| 0.021813 | 0.013651 | AE |
| 0.022148 | 0.01998 | AE |
| 0.022408 | 0.017775 | AE |
| 0.022444 | 0.004138 | AE |
| 0.022722 | 0.053096 | AE |
| 0.022998 | 0.022815 | AE |
| 0.026014 | 0.019193 | AE |
| 0.026341 | 0.014986 | AE |
| 0.026612 | 0.025371 | AE |
| 0.027202 | 0.39711 | X-ray |
| 0.027472 | 0.73949 | X-ray |
| 0.030134 | 0.00794 | AE |
| 0.030553 | 0.094917 | IC |
| 0.03088 | 0.008609 | IC |
| 0.030944 | 0.071879 | X-ray |
| 0.030995 | 0.13976 | X-ray |
| 0.03115 | 0.003316 | IC |
| 0.031237 | 0.001442 | X-ray |
| 0.03171 | 0.042891 | X-ray |
| 0.034673 | 0.021342 | IC |
| 0.035492 | 0.005052 | IC |
| 0.035492 | 0.066476 | Gamma |

| **^123^I** | | |
| --- | --- | --- |
| **E_i_** (MeV) | **n_i_** | **Transition** |
| 5.554E-05 | 0.37273 | AE |
| 0.0006991 | 1.8005 | AE |
| 0.0029555 | 0.38805 | AE |
| 0.0032261 | 0.16809 | AE |
| 0.0035533 | 0.050512 | AE |
| 0.0037197 | 0.19899 | AE |
| 0.0037693 | 0.033679 | X-ray |
| 0.0039903 | 0.081921 | AE |
| 0.0040295 | 0.021713 | X-ray |
| 0.004252 | 0.028269 | AE |
| 0.0043016 | 0.006062 | X-ray |
| 0.0043175 | 0.030176 | AE |
| 0.0045226 | 0.011595 | AE |
| 0.0048498 | 0.004215 | AE |
| 0.021813 | 0.008474 | AE |
| 0.022148 | 0.012403 | AE |
| 0.022408 | 0.011034 | AE |
| 0.022444 | 0.002569 | AE |
| 0.022722 | 0.03296 | AE |
| 0.022998 | 0.014163 | AE |
| 0.026014 | 0.011914 | AE |
| 0.026341 | 0.009303 | AE |
| 0.026612 | 0.015749 | AE |
| 0.027202 | 0.24651 | X-ray |
| 0.027472 | 0.45905 | X-ray |
| 0.030134 | 0.004929 | AE |
| 0.030944 | 0.044619 | X-ray |
| 0.030995 | 0.08676 | X-ray |
| 0.031237 | 0.000895 | X-ray |
| 0.03171 | 0.026625 | X-ray |
| 0.12716 | 0.13618 | IC |
| 0.15403 | 0.016145 | IC |
| 0.15436 | 0.001165 | IC |
| 0.15463 | 0.00042 | IC |
| 0.15815 | 0.003519 | IC |
| 0.15897 | 0.833 | Gamma |
| 0.15897 | 0.00084 | IC |
| 0.24796 | 0.000711 | Gamma |
| 0.28103 | 0.000791 | Gamma |
| 0.34635 | 0.001258 | Gamma |
| 0.44002 | 0.004282 | Gamma |
| 0.49715 | 9.97E-05 | IC |
| 0.50533 | 0.003157 | Gamma |
| 0.52896 | 0.013911 | Gamma |
| 0.53854 | 0.003815 | Gamma |
| 0.62457 | 0.000833 | Gamma |
| 0.68795 | 0.000267 | Gamma |
| 0.73578 | 0.000616 | Gamma |
| 0.78359 | 0.000594 | Gamma |

| **^103^Pd** | | |
| --- | --- | --- |
| **E_i_ (MeV)** | **n_i_** | **Transition** |
| 0.000397 | 1.724 | AE |
| 0.0022139 | 0.42661 | AE |
| 0.0023562 | 0.16284 | AE |
| 0.002622 | 0.027415 | AE |
| 0.0026953 | 0.18592 | AE |
| 0.0026968 | 0.020096 | X-ray |
| 0.0028376 | 0.069925 | AE |
| 0.0028344 | 0.01253 | X-ray |
| 0.0029998 | 0.02215 | AE |
| 0.0031034 | 0.013845 | AE |
| 0.0031421 | 0.008811 | AE |
| 0.016297 | 0.010159 | AE |
| 0.016564 | 0.016757 | AE |
| 0.016707 | 0.013533 | AE |
| 0.016806 | 0.003679 | AE |
| 0.016955 | 0.049533 | AE |
| 0.017104 | 0.019536 | AE |
| 0.019285 | 0.014716 | AE |
| 0.019551 | 0.011659 | AE |
| 0.019693 | 0.020149 | AE |
| 0.020074 | 0.1981 | X-ray |
| 0.020216 | 0.37518 | X-ray |
| 0.022201 | 0.005796 | AE |
| 0.022699 | 0.034855 | X-ray |
| 0.022724 | 0.066032 | X-ray |
| 0.023172 | 0.017446 | X-ray |
| 0.03007 | 4.97E-07 | IC |
| 0.03919 | 1.19E-05 | IC |
| 0.049878 | 5.62E-08 | IC |
| 0.058998 | 1.35E-06 | IC |
| 0.059264 | 9.08E-08 | IC |
| 0.061914 | 2.73E-07 | IC |
| 0.06241 | 5.32E-08 | IC |
| 0.06241 | 1.04E-05 | Gamma |
| 0.27176 | 4.62E-07 | IC |
| 0.29157 | 5.18E-08 | IC |
| 0.29448 | 1.01E-08 | IC |
| 0.29498 | 2.8E-05 | Gamma |
| 0.33423 | 3.02E-06 | IC |
| 0.35404 | 3.15E-07 | IC |
| 0.3543 | 4.34E-08 | IC |
| 0.35445 | 3.91E-08 | IC |
| 0.35695 | 7.4E-08 | IC |
| 0.35745 | 1.42E-08 | IC |
| 0.35745 | 0.000221 | Gamma |
| 0.47386 | 1.8E-07 | IC |
| 0.49367 | 2E-08 | IC |
| 0.49708 | 3.96E-05 | Gamma |

| **^177^Lu** | | | | | | | | |
| --- | --- | --- | --- | --- | --- | --- | --- | --- |
| **E_i_ (MeV)** | **n_i_** | **Transition** | **E_i_ (MeV)** | **n_i_** | **Transition** | **E_i_ (MeV)** | **n_i_** | **Transition** |
| 1.943E-05 | 1.28087 | X-ray | 0.009553 | 2.24E-05 | X-ray | 0.065047 | 0.000684 | X-ray |
| 3.314E-05 | 8.927E-08 | X-ray | 0.009576 | 7.95E-06 | X-ray | 0.065106 | 0.001323 | X-ray |
| 0.0001205 | 1.235E-07 | X-ray | 0.01028 | 0.00186 | AE | 0.065256 | 1.45E-05 | X-ray |
| 0.0002199 | 9.092E-05 | X-ray | 0.010387 | 2.49E-06 | X-ray | 0.065436 | 0.000154 | X-ray |
| 0.000278 | 0.048 | AE | 0.010537 | 0.002114 | X-ray | 0.065444 | 0.00032 | X-ray |
| 0.000482 | 1.052E-07 | X-ray | 0.01069 | 1.48E-05 | X-ray | 0.065476 | 1.61E-07 | X-ray |
| 0.001639 | 0.0038861 | X-ray | 0.010725 | 5.31E-07 | X-ray | 0.065477 | 1.93E-07 | X-ray |
| 0.0016867 | 1.052E-05 | X-ray | 0.010736 | 4.81E-06 | X-ray | 0.069547 | 4.85E-05 | IC |
| 0.00189 | 0.1925 | AE | 0.010757 | 2.29E-05 | X-ray | 0.071243 | 0.000268 | IC |
| 0.006164 | 0.0010975 | IC | 0.01081 | 4.71E-05 | X-ray | 0.071646 | 1.34E-05 | IC |
| 0.00698 | 0.019 | AE | 0.0108 | 0.000182 | AE | 0.071646 | 0.00154 | Gamma |
| 0.0069823 | 0.0008755 | X-ray | 0.010869 | 6.39E-05 | X-ray | 0.101705 | 0.005528 | IC |
| 0.0072002 | 7.145E-06 | X-ray | 0.011019 | 7.37E-07 | X-ray | 0.102187 | 0.03474 | IC |
| 0.0074593 | 6.497E-06 | X-ray | 0.01103 | 1.15E-06 | X-ray | 0.103392 | 0.030678 | IC |
| 0.00751 | 0.0016 | AE | 0.011199 | 6.73E-06 | X-ray | 0.110851 | 0.017713 | IC |
| 0.00765 | 0.0199 | AE | 0.011207 | 8.82E-06 | X-ray | 0.11295 | 0.004816 | IC |
| 0.0078328 | 0.0014487 | X-ray | 0.011239 | 7.53E-09 | X-ray | 0.11295 | 0.064 | Gamma |
| 0.0078896 | 0.0127841 | X-ray | 0.01124 | 1.12E-08 | X-ray | 0.12548 | 3.04E-05 | IC |
| 0.00883 | 0.0115 | AE | 0.0425 | 0.000248 | AE |  |  |  |
| 0.008187 | 0.0003414 | X-ray | 0.0431 | 0.000362 | AE |  |  |  |
| 0.008664 | 9.878E-06 | X-ray | 0.0436 | 0.000049 | AE |  |  |  |
| 0.0088869 | 0.0001939 | X-ray | 0.0442 | 0.000256 | AE |  |  |  |
| 0.0090326 | 0.0001536 | X-ray | 0.046 | 0.000239 | AE |  |  |  |
| 0.0090375 | 0.0117562 | X-ray | 0.047467 | 0.051707 | IC |  |  |  |
| 0.0091 | 0.0033 | AE | 0.0481 | 0.00054 | AE |  |  |  |
| 0.0091231 | 1.44E-06 | X-ray | 0.0519 | 0.000309 | AE |  |  |  |
| 0.009146 | 0.00025 | X-ray | 0.0524 | 0.000229 | AE |  |  |  |
| 0.0091826 | 1.409E-06 | X-ray | 0.0536 | 0.00037 | AE |  |  |  |
| 0.0093327 | 0.000248 | X-ray | 0.054719 | 0.016317 | X-ray |  |  |  |
| 0.0093436 | 0.0022216 | X-ray | 0.055924 | 0.028549 | X-ray |  |  |  |
| 0.00936 | 0.00114 | AE | 0.060401 | 0.000119 | IC |  |  |  |
| 0.0094858 | 3.063E-05 | X-ray | 0.060883 | 4.14E-05 | IC |  |  |  |
| 0.009512 | 3.131E-07 | X-ray | 0.061 | 0.000157 | AE |  |  |  |
| 0.0095195 | 5.309E-06 | X-ray | 0.062088 | 5.1E-05 | IC |  |  |  |
| 0.0095202 | 2.878E-07 | X-ray | 0.063124 | 0.003039 | X-ray |  |  |  |
| 0.0095311 | 9.737E-07 | X-ray | 0.063383 | 0.005874 | X-ray |  |  |  |
| 0.0095329 | 5.467E-06 | X-ray | 0.063757 | 6.17E-05 | X-ray |  |  |  |
| 0.0095525 | 2.628E-06 | X-ray | 0.063813 | 7.85E-05 | X-ray |  |  |  |

**Supplementary Figure 16.** ^177^Lu β full spectrum continuous curve source in MIRD-Tab.

The ^177^Lu full spectrum table, shows all transitions except β electron Full spectrum, cause is a continuous curve source data in MIRD-Tab continuous histogram source data in MIRDcell that shown in supplementary figure 16 for MIRD-Tab data and figure 17 for MIRDcell data center.

**Supplementary Figure 17.** ^177^Lu β full spectrum continuous histogram source in MIRDcell.

**Referrences:**

1.	Eckerman KF, Endo A, eds. MIRD Radionuclide Data and Decay Schemes. Reston, VA: Society of Nuclear Medicine and Molecular Imaging; 2008.

2.	Goddu SM, Howell RW, Bouchet LG, Bolch WE, Rao DV. MIRD Cellular S Values: Self-Absorbed Dose per Unit Cumulated Activity for Selected Radionuclides and Monoenergetic Electron and Alpha Particle Emitters Incorporated into Different Cell Compartments. Reston, VA: Society of Nuclear Medicine; 1997.

3.	Vaziri B, Wu H, Dhawan AP, Du P, Howell RW, Bolch WE, et al. MIRD pamphlet No. 25: MIRDcell V2.0 software tool for dosimetric analysis of biologic response of multicellular populations. Journal of Nuclear Medicine. 2014 Sep 1;55(9):1557–64.

4.	Howell R W. Radiation spectra for Auger-electron emitting radionuclides: report no. 2 of AAPM Nuclear Medicine Task Group No. 6. Med. Phys. 1992.

5.	OpenGATE collaboration : http://www.opengatecollaboration.org • OpenGATE spokesperson: L. Maigne (LPC UMR 6533 CNRS/IN2P3 CFF• O technical coordinator: DS (CREATIS UC 5220, LF• G authors: authors. GATE Documentation. OpenGATE Collaboration; 2022.

6.	Falzone N, Fernández-Varea JM, Flux G, Vallis KA. Monte Carlo evaluation of Auger electron-emitting theranostic radionuclides. Journal of Nuclear Medicine. 2015 Sep 1;56(9):1441–6.

7.	Bousis C, Emfietzoglou D, Hadjidoukas P, Nikjoo H. Monte Carlo single-cell dosimetry of Auger-electron emitting radionuclides. Phys Med Biol. 2010;55(9):2555–72.

8.	Seifi Moradi M, Shirani Bidabadi B. Micro-dosimetry calculation of Auger-electron-emitting radionuclides mostly used in nuclear medicine using GEANT4-DNA. Applied Radiation and Isotopes. 2018 Nov 1;141:73–9.
